# First Identification of Fig Virus A and Fig Virus B in *Ficus carica* in Italy

**DOI:** 10.3390/plants12071503

**Published:** 2023-03-29

**Authors:** Serafina Serena Amoia, Michela Chiumenti, Angelantonio Minafra

**Affiliations:** 1 Institute for Sustainable Plant Protection (IPSP)—National Research Council, 70126 Bari, Italy; 2 Department of Soil, Plant and Food Sciences, University of Bari Aldo Moro, 70121 Bari, Italy

**Keywords:** *Ficus carica*, double-stranded RNA, high throughput sequencing, virus detection, closteroviruses

## Abstract

Extracts of double-stranded RNA from three fig cultivars from an Apulian (Southern Italy) germplasm collection were used for high-throughput sequencing and revealed the presence of two distinct, recently described closteroviruses. Sequences obtained from these Apulian isolates belong to fig virus A and fig virus B and cover 38 and 25% of their RNA genome, respectively. Primer sets designed on selected contigs confirmed the presence of each virus in infected plants. A close phylogenetic relationship, investigated in a fragment of HSP70h protein, occurs among these isolates and the reference genomes. A nucleotide divergence (ranging from 10 to 30% along the different genes) was observed among our isolates and the reference genomes. This is the first finding of these virus species in autochthonous fig accessions in Europe.

## 1. Introduction

Domestication of fig trees (*Ficus carica* L.) is considered one of the oldest agricultural practices in the Mediterranean basin [1]. The propagation of this species by self-rooting or grafting led to a widespread presence of several virus-like diseases in fig germplasm [2], to which only recently different viruses and viroids have been associated. The viruses described to date in fig trees belong to an array of taxa [3], with many of them belonging to the family *Closteroviridae* [4].

The long persistence of those perennial trees in the field facilitates mixed infections due to repeated exposure to virus vectors (mealybugs, aphids, mites). The combination of different co-infecting virus species brings forth an extraordinary range of symptoms (on affected leaves, shoots and fruits) of the condition commonly as known ‘mosaic disease’ [5]. The key agent of the disease was definitively recognized in the mite-transmitted fig mosaic virus (FMV, genus *Emaravirus* [6]).

The degraded sanitary status of currently used fig varieties often implies yield losses of an unacceptable level for sustainable cultivation. To avoid the spread of regulated viruses (Regulations EU 2019/2072 and 2018/2019), a generic diagnostic tool to screen the propagation material is needed. High-throughput sequencing (HTS) demonstrates enough sensitivity for the detection of known and the de novo discovery of unknown systemic pathogens in certification programs of vegetatively propagated crops [7,8,9]. Indeed, the application of HTS occurred recently in the simultaneous and independent identification and complete genome description of two novel closteroviruses in different fig accessions in Argentina (only deposited in GenBank) and Japan [10]—namely, fig virus A (FiVA) and fig virus B (FiVB). The characterization of the phytosanitary status of autochthonous fig cultivars in Apulia, Italy, led to the identification of isolates of the same two virus species using different HTS platforms. This finding sheds a light on the long-term coexistence of these viruses within the host and sets up further studies about their biology and phylogenetic relationships.

## 2. Results and Discussion

For the Illumina-indexed libraries, an average of 28 million reads was achieved, with more than a million passed reads (mean quality score: 30.16) per barcode inside each library. Several independent, small-size contigs, obtained from Illumina libraries in 2018, were sorted into two distinct groups; these groups were selectively mapped to members of the genus *Closterovirus* with a variable identity degree, albeit below the species demarcation threshold [4]. The evidence that those accessions separately contained two different viruses was further explored by designing specific primers using the Primer3 web version 4.1.0 online tool. A list of nanopore-obtained reads and main Illumina-derived contigs is reported in Supplementary Table S1, where the sequences selected for the primer design and RT–PCR confirmation are marked. All the designed primers are presented in Table 1.

Total RNA extracted by VdN, VCC and Ric plants was then analyzed by RT–PCR amplification. Some of the DNA fragments amplified from primer sets of two Illumina contigs per each accession, and from one read of the VCC nanopore barcoded library, were cloned in a plasmid vector and conventionally sequenced (see Supplementary Table S1). Since the Ric source tested positive, with the expected sized amplicons, to the primer sets designed on the VdN contigs and not with those designed on VCC ones, these PCR results were sufficiently specific that the Ric amplicons were not cloned or sequenced.

A phylogenetic relationship among selected sequences produced in this study and similar regions of other closteroviruses from the GenBank repository was evaluated. An overlapping portion of 72 amino acids in the partial HSP70h translated proteins of the two related viruses was found in VdN contig 59184 and VCC contig 62228. Sequences of the related viruses, spanning the same borders of the VCC/VdN overlapping fragments, were aligned using the MUSCLE algorithm [11] and the best substitution model prediction was obtained (LG + G). A maximum likelihood phylogenetic tree was inferred in the MEGA X program [12] with 1000 replicates of bootstrap.

In the case of Illumina libraries, due to the enriched virus-derived dsRNA fraction that was used as a template for sequencing, a proportion of the assembled contigs belonging to already known fig viruses (such as fig badnavirus 1, fig mosaic emaravirus and fig mild mottle–associated virus) were identified.

When nanopore-derived reads became available from our samples in 2021, these sequences were used to conduct a new BlastN database interrogation. We gained evidence that the closterovirus-like sequences previously found in those accessions should be attributed to a couple of recently described closterovirus species. These were fig virus A (acc. nr. MN817232, the virus isolate present in VCC) and fig virus B (acc. nr. MN817233, the virus isolates infecting VdN and Ric). This finding was confirmed by the re-analysis of the Illumina contigs.

A similar homology between the virus sequences analyzed in our experiment and the fig viruses A and B (analyzed in Korea by Park et al. (2021) [10] from a Japanese transcriptome dataset [13]) was mirrored, with two other viruses (fig closterovirus 1 and fig closterovirus 2; acc. nrs. MW489855.1, MW489856.1, respectively) sharing a nucleotide identity around 99% with each respective homologous. The full genomic sequences of the latter isolates were only deposited in GenBank with no additional information.

**Figure 1:**
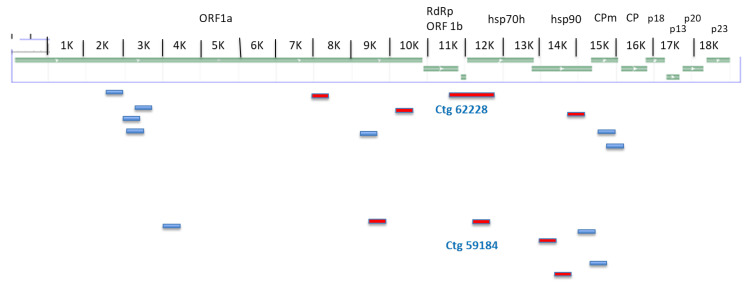
A graphic representation of the distribution, along a generalized closterovirus genome, of the contigs obtained in the two Illumina sequenced libraries for VdN and VCC accessions. ORFs names are indicated above the genome: RdRp: RNA-dependent RNA polymerase; hsp: Heat Shock Protein; CPm: Coat Protein minor; p: protein. Only the major contigs are reported; the size is not in scale. Contigs used for the primer design and RT–PCR validation on fig RNA extracts are in red. The codes for the two contigs (VCC 62228 and VdN 59184) overlapping at the 5′ of HSP70h gene are reported.

A selected array of Illumina-derived contigs from VCC and VdN barcoded libraries, having a significant length (generally more than 100 nt) and showing homology with the closer related viruses through Blast search, was listed in Supplementary Table S1. For those contigs, the depth of redundant reads per position, the nucleotide identity with the related virus and the genome position on the same hit were also reported. The identity value was variable according to the involved genes. A nucleotide identity ranging from 74 to 90% was observed for VdN contigs, whereas a range of 70 to 84% was observed for VCC contigs. The lowest identity value was expressed by the VCC contig 15991: it has 70% nucleotide and 75% amino acid identity with the related fig closterovirus 2; the same sequence shows a 67.6% nt identity with the other heterologous virus (fig closterovirus 1/FiVB). The horizontal coverage along the genomes, summing up all the attributed and non-overlapping Illumina contigs (not all of them exhaustively listed in Supplementary Table S1), was around 38% (7450 nt) for the VCC isolate and 25% (4840 nt) for VdN, respectively. The scattered localization of those assembled fragments, derived from the random-primed cDNA reads on the denatured dsRNA templates, is represented in Figure 1 over a closterovirus model genome.

A different picture is drawn by nanopore sequencing. In the bulk of 4.3 × 10^6^ passed reads (with a median read length of 315 nt) for the eight different barcodes mixed in the single run, the percentage of total reads from the three fig barcodes gave a similar representation (12.6% for VdN, 16.8% for Ric, 14% for VCC, respectively). However, a few reads in the single fig–derived barcoded sequences could be identified by mapping against the investigated virus reference genomes. No assembly could be produced because of the scattered position of the reads along the genomes; however, these reads were large enough to be univocally annotated by BlastN/X. The error rate of the nanopore method, higher than Illumina sequencing, had the major drawback of hampering the precise reconstruction of the coding frame inside the few obtained reads.

For the Ric accession, three independent virus reads were identified when annotated by BlastN: two were overlapping at the very 5′ portion of the genome (Ric b1f6bb93 and Ric d08096f4), while a third one was located almost in the middle (Ric e739c51c). Conversely, only one read was observed for VCC (VCC read 12eb-c33) and two for VdN (VdN bb4768af, VdN 191db814) (Supplementary Table S1). All the primer sets designed on VCC contigs and reads were able to amplify a true-size band from the same accession and three amplicons were cloned and sequenced (from contigs 15591, 23,135 and 68710) showing an identity, with sequences from the respective contigs being higher than 98%. The cloned sequence derived from VCC nanopore read 12eb-c33 primers matched at 82.3% with the related read sequence, while the identity reached 89% when the cDNA-derived amplicon sequence was blasted; this confirmed a less precise base calling of the nanopore library.

All the primers designed on VdN Illumina contigs and nanopore reads correctly amplified both VdN and Ric cDNAs, demonstrating that isolates of the same virus were present in both accessions. VdN cloned sequences mapped with the original VdN contigs, with an identity ranging from 95.3 to 98.8%. While no PCR cross-reactivity was detected with specific primers between the two groups (VCC vs. VdN/Ric and viceversa), the cloned sequences generally recognized—when blasted—the heterologous virus at a lower extent than the homologous virus; this was due to the high similarity at the whole genome level (56.7% nt identity) between the two viruses (i.e., FiVA and FiVB).

**Figure 2:**
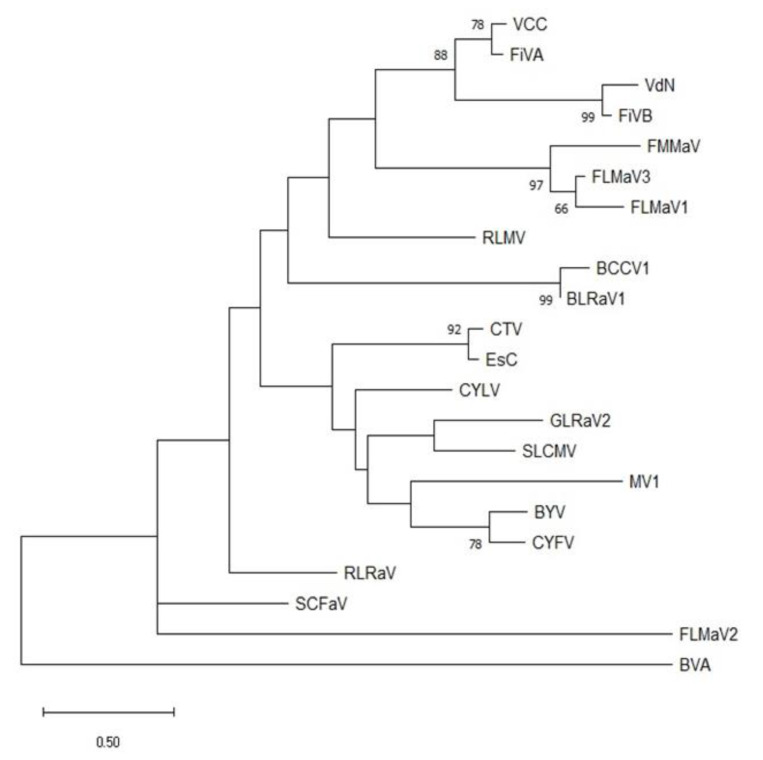
Maximum likelihood phylogenetic reconstruction of the 5′ portion of 72 amino acids in the HSP70h protein of selected closterovirids. The ampeloviruses FLMaV2 and BVA, unassigned species in the family *Closteroviridae*, represent the outgroups. Bootstrap values higher than 60% on supported nodes and a distance bar are shown. Used accession numbers for the virus proteins are: BCCV1: blackcurrant closterovirus 1 (AYA2226); BcLRaV1: blackcurrant leafroll–associated virus 1 (YP009553662); BVA: bluberry virus A (QYU71616); BYV: beet yellow virus (NP041872); CTV: Citrus tristeza virus (AYA60225); CYFV: carnation yellow fleck virus (YP008858553); CYLC: carrot yellow leaf virus (YP003075968); EsC: Elephantopus scaber closterovirus (QJZ28396); FiVA: fig virus A (QN529663); FiVB: fig virus B (QN529677); FLMaV1: fig leaf mottle-associated virus 1 (CAJ34535); FLMaV2: fig leaf mottle-associated virus 2 (AC575347); FLMaV3: fig leaf mottle-associated virus 3 (AKM77648); FMMaV: fig mild mottle-associated virus (ACU57193); GLRaV2: grapevine leafroll-associated virus 2 (ACE79591); MV1: mint virus 1 (AAX98726); RLMV: raspberry leaf-mottle virus (AAY82288); RLRaV: rose leaf rosette-associated virus (QQ202304); SCFaV: strawberry chlorotic fleck–associated virus (YP762625); SLCMV: soybean leaf crinkle mottle virus (BCR37034); VdN: Verde di Natale; VCC: Vito Carlo Casamassima.

To investigate the phylogenetic relationship of the Apulian FiVA and FiVB isolates, a small stretch at 5′ of hsp70 protein could be used since only two contigs (VCC_62228 and VdN_59184) overlapped in this region and could be compared with the aligned proteins of other members in the genus *Closterovirus*. The derived bootstrapped dendrogram (Figure 2) is in agreement with what is shown in Park et al. (2021) [10]. Fig leaf mottle-associated virus 2 (FLMaV-2; genus *Ampelovirus*) and blueberry virus A (BVA; unassigned species in the family *Closteroviridae*) behave as outgroups. VCC and VdN isolates, each one closer to the homologous virus in a specific node, belong to a phylum formed by two fig-infecting closteroviruses (a predictive single lineage including fig mild mottling-associated virus (FMMaV): fig leaf mottle-associated virus 1 and 3 (FLMaV 1 and 3). This feature may support the hypothesis that an ancestral closterovirus was at the origin of the currently observed species in fig. This cluster thus shares minimal distance and a common ancestor node with those viruses (raspberry leaf mottle virus (RLMV) and blackcurrant closterovirus 1 (BCCV-1)) that occur with major identity scores when many independent contigs, along the whole genome of VCC or VdN, are BlastN annotated.

The discovery of these isolates of FiVA and FiVB in local fig germplasm (so far, the first time identified reported in Europe) indicates a potentially widespread distribution of these viruses. A genetic divergence of these isolates from those described in South America and Asia, and their relative genetic closeness, is also remarkable. The identified viruses share a co-infection condition with other viral pathogens in affected fig trees and, consequently, no association with specific symptoms could be attributed to these two viruses. Therefore, it was worthwhile to develop molecular tools for their detection in the aim of research and containment purposes.

## 3. Material and Methods

A single accession of each Apulian autochthonous fig cultivar (Verde di Natale (VdN), Vito Carlo Casamassima (VCC) and Ricotta (Ric)) showing mosaic disease symptom, was collected in 2014—in the frame of a Regional Project on the preservation of crop biodiversity—in different locations in Apulia and kept self-rooted in a collection plot at CRSFA (Locorotondo, Italy). In 2018, to check the global sanitary status of the germplasm collection, leaf tissues (up to 500 mg) were collected from each tree, and total nucleic acid was extracted with guanidine isothiocyanate buffer [14]. The dsRNA fraction of the extract was purified by affinity through two chromatography steps on CC-41 cellulose (Whatman, Fisher Scientific, Leicestershire, UK). Up to 10 different dsRNA extracts, each one from a single accession (from fig and other fruit trees, such as cherry, apricot and apple), were then pooled to be run as a single library; they extracts were approximately in the same amount according to their OD concentration. Two libraries for Illumina HTS were prepared as in Marais et al. (2018) [15]. The first library included the cDNA of the VdN-extracted dsRNA, while the second one included the cDNA derived from the VCC accession. A paired-end sequencing (2 × 150-nt-long reads) of the libraries was performed by an external service (Genewiz, Leipzig, Germany).

In 2021, a new dsRNA extraction from the same two fig accessions, and an additional one from the Ric source, were performed using the protocol described above. Every single dsRNA was separately heat denatured and reverse transcribed for the synthesis of cDNA-PCR barcoded libraries, according to the SQK-PCB109 cDNA-PCR sequencing kit (Oxford Nanopore Technologies [ONT], Oxford, UK) and using a nonamer anchored primer (5′-ACTTGCCTGTCGCTCTATCTTCNNNNNNNNN-3′) [16] and the Maxima H-Minus Reverse Transcriptase (Thermo Fisher Scientific, USA). The three different barcoded libraries were equimolarly pooled and loaded as a single mixture onto the MinION flow cell (Flo-Min 106d r9.4.1) for a 7 h run. The generated electrical signals were base called in real-time using the standard MinKNOW software (v. 19.06.9 Oxford Nanopore Technologies, Oxford, UK).

Illumina libraries were checked for their quality using the fastxtoolkit (http://hannonlab.cshl.edu/fastx_toolkit/ (accessed on 27 February 2023)), keeping only reads with a Phred score higher than 20 and adapter trimmed. Such reads were then de novo assembled in contigs using Spades v.3.15.3 [17] with k-mers ranging from 51 to 121. De novo assembled contigs (Illumina, San Diego, CA, USA) and quality filtered reads (nanopore) were annotated using nt database or viral sequence custom database via BLASTn and BLASTx algorithms, respectively. Blast results were considered significant when the e-value thresholds were 10^−6^ and 10^−4^, respectively, for BLASTn and BLASTx outputs. Bowtie software v.2.3.5.1 [18] and Minimap2 [19] were used for the alignment of Illumina and Nanopore reads, respectively, with the identified viral sequences.

## Figures and Tables

**Table 1:** List of primers designed in the selected contigs/reads obtained from VCC and VdN accessions, together with their amplicon size. The percentage of nt identity of the cloned and sequenced amplicons with the original sequences from libraries is reported in brackets.

Isolate	Contig OR Read	Forward	Reverse	Amplicon Size (bp)	Sequenced Clone
**VCC**	Contig 15991	CCCTTGCAACCTCCTAGAGA	GTTGTCCCAAACGGCGTAAT	201	Yes (98.54%)
	Contig 23135	TGATGTCTTCTTTGGCCGAC	CGTGTCCTCTTCTTTCCCCT	243	Yes (100%)
	Contig 68710	CCAGGGTTTGCGGCTTTTAA	GCTCAAGAGTTCGTTGTGGA	158	No
	Contig 62228	TGCGATTTTCCGATCAATGAAG	ATGGTCATGCGGCTGAATTG	160	No
	Minion read 12eb c33	TTGCCAGCACATTATTCGTGGCT	CTAATCGTAGAACTAAATCTCT	180	Yes (82.3%)
**VdN**	Contig 33687	CACTTTCTCTACGTTCGTGGT	CCCAGCGGACTATTCATATCTAT	214	Yes (98.83%)
	Contig 72524	CAAAAACAGTGCGAGTTCCCG	ATGACATTCGCAAATTTCCACT	187	Yes (95.38%)
	Contig 59148	AATGAAATACGGCTTTGATGCT	TAAGTTGCGAGCGTTACACC	103	No
	Contig 29148	TCGTGTCGATGCAAGAAGTC	GCTTGCACCCAGACTACCTA	101	No
	Minion read 191db814	CCGCCTTTCATYCAGTCCYTC	GCGACGATCGCGCAAAAGCGC	200	No

## Data Availability

The partial sequences of VCC and VdN isolate of FiVA and FiVB respectively has been deposited in GenBank (Acc. nr. ON959379, ON959380, ON959381, ON938193, ON939595, ON939596, ON939597, ON939598). All the sequencing output dataset generated in the study is freely available upon request to the Corresponding Author.

## Supplementary Materials

The following supporting information can be downloaded at: https://www.mdpi.com/article/10.3390/plants12071503/s1; Supplementary Table S1: Major contigs/reads obtained from the three fig accessions.

## References

[1] Kislev M.E., Hartmann A., Bar-Yosef O. (2006). Early Domesticated Fig in the Jordan Valley. Science.

[2] Minafra A., Savino V., Martelli G.P. (2017). Virus Diseases of Fig and Their Control. Acta Hortic..

[3] Preising S., Borges D.F., de Queiroz Ambrósio M.M., da Silva W.L. (2021). A Fig Deal: A Global Look at Fig Mosaic Disease and Its Putative Associates. Plant Dis..

[4] Fuchs M., Bar-Joseph M., Candresse T., Maree H.J., Martelli G.P., Melzer M.J., Menzel W., Minafra A., Sabanadzovic S., Report Consortium I. (2020). ICTV Virus Taxonomy Profile: Closteroviridae. J. Gen. Virol..

[5] Condit I.J., Horne W.T. (1933). A mosaic of the fig in California. Phytopathology.

[6] Elbeaino T., Digiaro M., Alabdullah A., De Stradis A., Minafra A., Mielke N., Castellano M.A., Martelli G.P. (2009). A Multipartite Single-Stranded Negative-Sense RNA Virus Is the Putative Agent of Fig Mosaic Disease. J. Gen. Virol..

[7] Al Rwahnih M., Daubert S., Golino D., Islas C., Rowhani A. (2015). Comparison of Next-Generation Sequencing Versus Biological Indexing for the Optimal Detection of Viral Pathogens in Grapevine. Phytopathology.

[8] Maliogka V.I., Minafra A., Saldarelli P., Ruiz-García A.B., Glasa M., Katis N., Olmos A. (2018). Recent Advances on Detection and Characterization of Fruit Tree Viruses Using High-Throughput Sequencing Technologies. Viruses.

[9] Olmos A., Boonham N., Candresse T., Gentit P., Giovani B., Kutnjak D., Liefting L., Maree H.J., Minafra A., Moreira A. (2018). High-Throughput Sequencing Technologies for Plant Pest Diagnosis: Challenges and Opportunities. EPPO Bull..

[10] Park D., Goh C.J., Hahn Y. (2021). Two Novel Closteroviruses, Fig Virus A and Fig Virus B, Identified by the Analysis of the High-Throughput RNA-Sequencing Data of Fig (*Ficus Carica*) Latex. Acta Virol..

[11] Edgar R.C. (2004). MUSCLE: Multiple Sequence Alignment with High Accuracy and High Throughput. Nucleic Acids Res..

[12] Kumar S., Stecher G., Li M., Knyaz C. (2018). Tamura MEGA X: Molecular Evolutionary Genetics Analysis across Computing Platforms. Mol. Biol Evol..

[13] Kitajima S., Aoki W., Shibata D., Nakajima D., Sakurai N., Yazaki K., Munakata R., Taira T., Kobayashi M., Aburaya S. (2018). Comparative Multi-Omics Analysis Reveals Diverse Latex-Based Defense Strategies against Pests among Latex-Producing Organs of the Fig Tree (*Ficus Carica*). Planta.

[14] Foissac X., Svanella-Dumas L., Gentit P., Dulucq M.-J., Marais A., Candresse T. (2005). Polyvalent Degenerate Oligonucleotides Reverse Transcription-Polymerase Chain Reaction: A Polyvalent Detection and Characterization Tool for Trichoviruses, Capilloviruses, and Foveaviruses. Phytopathology.

[15] Marais A., Faure C., Bergey B., Candresse T., Pantaleo V., Chiumenti M. (2018). Viral Double-Stranded RNAs (DsRNAs) from Plants: Alternative Nucleic Acid Substrates for High-Throughput Sequencing. Viral Metagenomics: Methods and Protocols.

[16] Della Bartola M., Byrne S., Mullins E. (2020). Characterization of Potato Virus Y Isolates and Assessment of Nanopore Sequencing to Detect and Genotype Potato Viruses. Viruses.

[17] Bankevich A., Nurk S., Antipov D., Gurevich A.A., Dvorkin M., Kulikov A.S., Lesin V.M., Nikolenko S.I., Pham S., Prjibelski A.D. (2012). SPAdes: A New Genome Assembly Algorithm and Its Applications to Single-Cell Sequencing. J. Comput. Biol..

[18] Langmead B., Salzberg S.L. (2012). Fast Gapped-Read Alignment with Bowtie 2. Nat. Methods.

[19] Li H. (2018). Minimap2: Pairwise alignment for nucleotide sequences. Bioinformatics.

