# Supplementary material for: First Identification of Fig Virus A and Fig Virus B in Ficus carica in Italy
Source: Plants (Basel). 2023 Mar 29;12(7):1503. doi: 10.3390/plants12071503 (PMC10096765; doi:10.3390/plants12071503)
Supplement: Supplementary file 1 [file plants-12-01503-s001.zip › plants-2283171-supplementary.docx]

Brief report

First identification of fig virus A and fig virus B in *Ficus carica* in Italy

Serafina Serena Amoia^1,2^, Michela Chiumenti^1^ and Angelantonio Minafra^1^*

^1^ Institute for Sustainable Plant Protection (IPSP)—National Research Council, 70126 Bari, Italy.

^2^ Department of Soil, Plant and Food Sciences, University of Bari Aldo Moro, Bari, Italy.

***** Correspondence: angelantonio.minafra@ipsp.cnr.it

**Supplementary materials**

**Supplementary Table S1:** Major contigs/reads obtained from the three fig accessions, with position on the mapping genome and coverage depth. The first BlastN hit is reported with the % of nucleotide identity. (*): Virus-derived sequences used to design primers for amplification and cloning of RT-PCR products. The accession numbers are reported for the sequences deposited in GenBank. n/a: not applicable.

| **Contig/Read ID** | **Length** | **Position on 1^st^ BlastN hit genome** | **Nucleotide identity %** | **Coverage (sequence method)** | **Accession Number** |
| --- | --- | --- | --- | --- | --- |
| VdN read bb4768af | 204 | 249-454 (MW489855) | 84 | n/a (nanopore) |  |
| VdN read 191db814 | 249 | 214-470 (MW489855) | 82 | n/a (nanopore) |  |
| VdN 43964 | 582 | 7323-7904 (MW489855) | 87 | 10.826 (Illumina) | ON939598 |
| VdN 59148 | 244 | 11604-11844 (MW489855) | 83.8 | 9.702 (Illumina) |  |
| VdN 66917 | 188 | 18366-18572 (MW489855) | 82 | 4.079 (Illumina) |  |
| VdN 143131 | 124 | 2971-3094 (MW489855) | 87 | 2.656 (Illumina) |  |
| VdN 33687 | 275* | 14496-14756 (MW489855) | 87 | 21.213 (Illumina) | ON959381 |
| VdN 155712 | 62 | 8515-8592 (MW489855) | 74 | 1.677 (Illumina) |  |
| VdN 193893 | 71 | 2873-2965 (MW489855) | 90 | 2.084 (Illumina) |  |
| VdN 29148 | 177 | 14344-14493 (MW489855) | 87 | 17.830 (Illumina) |  |
| VdN 141012 | 98 | 2710-2829 (MW489855) | 89 | 3.020 (Illumina) |  |
| VdN 73953 | 70 | 8909-9000 (MW489855) | 90 | 4.385 (Illumina) |  |
| VdN 62366 | 175 | 7120-7316 (MW489855) | 87 | 6.428 (Illumina) |  |
| VdN 62837 | 109 | 6990-7118 (MW489855) | 83 | 4.366 (Illumina) |  |
| VdN 95695 | 81 | 12537-12639 (MW489855) | 85 | 2.666 (Illumina) |  |
| VdN 142124 | 156 | 3188-3365 (MW489855) | 89 | 2.608 (Illumina) |  |
| VdN 72524 | 476* | 8928-9457 (MW489855) | 85 | 6.295 (Illumina) | ON959381 |
| VCC read 12eb-c33 | 224* | 16641-16859 (MW489856) | 79 | n/a (nanopore) |  |
| VCC 39909 | 304 | 14772-15075 (MW489856) | 84 | 12.078 (Illumina) |  |
| VCC 68710 | 289 | 14489-14775 (MW489856) | 83 | 14.250 (Illumina) |  |
| VCC 131429 | 585 | 15238-15822 (MW489856) | 84 | 5.905 (Illumina) | ON938193 |
| VCC 139803 | 224 | 2825-3048 (MW489856) | 74 | 2.692 (Illumina) |  |
| VCC 62228 | 1426 | 11041-12468 (MW489856) | 80 | 9.292 (Illumina) | ON959379 |
| VCC 77014 | 556 | 12710-13265 (MW489856) | 82 | 4.403 (Illumina) | ON939595 |
| VCC 59285 | 321 | 8408-81117 (MW489856 | 78.4 | 5.316 (Illumina) |  |
| VCC 15991 | 697* | 7293-7981 (MW489856) | 70 | 9.554 (Illumina) | ON939596 |
| VCC 23135 | 1216* | 9456-10437 (MW489856) | 74 | 10.985 (Illumina) | ON939597 |
| VCC 74555 | 242 | 12467-12707 (MW489856) | 84 | 4.109 (Illumina) |  |
| VCC 171022 | 179 | 3780-3955 (MW489856) | 81 | 4.152 (Illumina) |  |
| Ric b1f6bb93 | 494 | 188 -693 (MW489855) | 79 | n/a (nanopore) |  |
| Ric d08096f4 | 330 | 125-453 (MW489855) | 80 | n/a (nanopore) |  |
| Ric e739c51c | 155 | 11502 - 11659 (MW489855) | 81 | n/a (nanopore) |  |
|  | | | | | |
